# Associations Between Pet Type (Co-Walkable, Indoor-Only, and Ornamental Pets) and Well-Being: Findings from a Large-Scale Cross-Sectional Study in Japan

**DOI:** 10.3390/ijerph22111654

**Published:** 2025-10-30

**Authors:** Kaori Endo, Anri Mutoh, Kazuya Ogawa, Miho Satoh

**Affiliations:** 1 Center for Preventive Medical Sciences, Chiba University, Chiba 263-8522, Japan; 2 Data Science Institute, Rissho University, Tokyo 141-8602, Japan; 3 Department of Fundamental Nursing, Graduate School of Medicine, Yokohama City University, Yokohama 236-0004, Japan

**Keywords:** companion animals, dogs, cats, fishes, quality of life, well-being, cross-sectional studies, Japan, housing, social determinants of health

## Abstract

The associations of pet ownership and well-being have been widely discussed, but previous studies have shown inconsistent results, often due to their limited sample size and diversity. We analyzed data from a nationally representative survey conducted by the Cabinet Office of Japan in 2019 (*n* = 10,293; age range = 15–89 years; 50.4% female). Ownership of co-walkable pets (e.g., dogs), indoor-only pets (e.g., cats), and ornamental pets (e.g., tropical fish) was examined as a predictor. Well-being was measured using eleven domains based on the OECD Better Life Index. Demographic covariates were included. Of the participants, 13.3% owned a co-walkable pet, 13.0% an indoor-only pet, and 6.8% an ornamental pet. The pet owners were more likely to live in a house they owned and have a larger floor area, higher income, and greater debt. The non-pet owners were more likely to live alone. In the unadjusted models, the ownership of co-walkable pets was associated with higher well-being in terms of housing and community. However, in the adjusted models, the ownership of co-walkable pets was associated with lower well-being in terms of income, jobs, environment, and, marginally, safety. No significant associations were found for indoor-only and ornamental pet ownership. In Japan, pet ownership requires both financial resources and adequate living space. It is also important to note that pet owners who go outside for walking their animals may also find that their environmental and economic circumstances are less satisfying.

## 1. Introduction

The association of pet ownership and subjective well-being has been widely discussed in both academic and public discourse. It is well-known that pet ownership has benefits for humans, including increased physical activity and reduced stress. For instance, it has been reported that dog owners engage in more walking [1], and that making eye contact with dogs can elevate oxytocin levels [2]. Furthermore, at a community level, pets have a positive effect on community health. Research in Australian cities indicates that pet owners exhibit stronger social connections among local residents [3]. Pet owners are more likely to get to know their neighbors, and dog owners in particular have been found to receive greater social support from friends they have met through their pets [4]. The benefits of pet ownership also extend to children and young people, who may experience increased self-esteem and reduced loneliness [5]. However, previous studies have yielded inconsistent findings. A recent systematic review highlighted the existence of conflicting results, noting substantial variation across studies in terms of definitions, study populations, and methodologies, which makes direct comparisons difficult and limits the ability to draw definitive conclusions [6]. Moreover, as noted in research on pet ownership and well-being [6,7], the literature in this field may be subject to publication bias, commonly referred to as the “file drawer effect.” This bias arises because the presumed positive effects of pet ownership are often emphasized, which may discourage the publication of studies reporting null or negative findings. To advance scientific understanding in this field, high-quality research using standardized measures and large, diverse populations is needed. In particular, studies that include non-Western populations and employ population-based survey data can enhance the generalizability and comparability of findings.

Previous studies have varied in how they treat pet ownership, with some analyzing pet ownership as a single variable regardless of the animal type and others stratifying their results by specific species [6,7]. The survey in the present study categorized pets based on the nature of interactions they involve, rather than by species alone. This approach resulted in three groups: co-walkable pets, indoor-only pets, and ornamental pets. Co-walkable pets, such as dogs, require regular physical activity. Their care often involves outdoor walks, which may strengthen bonds between the owner and pet, while also fostering social interactions with other pet owners or community members. Indoor-only pets, such as cats, are typically kept in enclosed spaces and do not require outdoor activity. Interaction tends to occur within the household, with the owner’s family members and other pets in the same house. A survey by the Japan Pet Food Association shows that, as of 2024, 83.5% of cats are kept indoors (https://petfood.or.jp/data-chart/, accessed on 21 October 2025). This figure appears to be higher than that reported in a previous study of Western regions (41.0% in Europe, the USA, Canada, Australia, and New Zealand) [8]. Ornamental pets, including tropical fish, do not involve physical contact or outdoor activity, as noted in a systematic review on fish’s effect on human well-being [9]. Nonetheless, they require dedicated care environments such as tanks or cages and may contribute to emotional regulation through visual observation and the calming effect of routine maintenance [10]. Especially in Japan—a non-Western country—goldfish (*Carassius auratus*) and fancy carp (*Cyprinus carpio*) have a long-standing history as ornamental pets [11,12]. Carp were first introduced from China in around the 1st century CE, and goldfish were imported from China in the early 16th century [11,12]. Initially, both species were primarily kept by the aristocrats and nobility. However, during the Edo period (1603–1868), they gradually spread widely among the common people, becoming popular as decorative pets and an established part of urban culture and life. Understanding how different types of pet ownership relate to multidimensional well-being in a non-Western country may inform globally generalizable urban planning, mental health policy, and housing strategies.

In addition, while previous studies on pet ownership and well-being have used a variety of mental health indicators (e.g., anxiety, depression, self-esteem, loneliness, quality of life, well-being, etc.) [5,6], the survey in this study used a scale that allows for multifaceted interpretation of well-being, OECD’s Better Life Index [13]. This influential index uses a multidimensional approach to assess well-being, considering social, economic, and environmental factors. It highlights the importance of considering societal priorities and individual preferences in well-being assessments. Well-being consists of multiple domains such as income, job satisfaction, environmental satisfaction, health perception, and more, each of which may be differentially associated with pet ownership.

The aim of this study is to clarify the association between different types of pet ownership and multiple domains of subjective well-being in a large, nationally representative sample in Japan. By doing so, we seek to provide robust evidence on whether and how pet ownership contributes to well-being in a non-Western context. For this purpose, using a large, nationally representative sample of over 10,000 individuals, we investigated the effects of owning different types of pets—co-walkable pets (e.g., dogs), indoor-only pets (e.g., cats), and ornamental pets (e.g., fish)—on eleven domains of well-being based on the OECD Better Life Index (e.g., income, jobs, housing, health, environment, community). By adjusting for a wide range of sociodemographic covariates, we aimed to reduce potential confounds and provide reliable evidence to elucidate whether pet ownership contributes meaningfully to well-being.

## 2. Materials and Methods

### 2.1. Data Source

We used data from the “Well-being Survey and Quality of life” study, conducted by the Cabinet Office of Japan between 25 January and 7 February 2019. The dataset was obtained through the Social Science Japan Data Archive (SSJDA), operated by the Center for Social Research and Data Archives, Institute of Social Science, The University of Tokyo. The SSJDA provides access to survey data for academic research purposes upon approval of a formal application, which requires submission of a research plan and a pledge to comply with data use policies. All data provided by SSJDA has been anonymized to ensure the protection of personal information. The survey targeted a nationally representative sample of Japanese residents aged 15 to 89 years. Non-probability quota sampling was employed. Participants were selected from a registered online panel and stratified by region, sex, and age group. A total of 10,293 valid responses were included in this analysis. Regional quotas were based on 7050 cases equally allocated across all 47 prefectures, with an additional 3243 cases proportionally allocated by population size (169 to 477 cases per prefecture). The participants were equally divided by gender (3525 men and 3525 women) across prefectures, with an additional 3243 participants allocated based on gender-specific population proportions. Age groups were also stratified, as follows: 15–24, 25–34, 35–44, 45–59, and 60–89 years. There were equal and proportionally allocated samples within each group. There were no missing responses in the dataset.

### 2.2. Measures

#### 2.2.1. Predictor: Pet Ownership

Pet ownership was assessed using the following items:

- Co-walkable pets (e.g., dogs): “Do you own a pet that can go outside for a walk, such as a dog?”
- Indoor-only pets (e.g., cats): “Do you own a pet that is kept only inside your home, such as a cat?”
- Ornamental pets (e.g., tropical fish): “Do you own a pet that is ornamental, such as a tropical fish?”

Each was coded as a binary variable (1 = yes, 0 = no).

#### 2.2.2. Outcome: Well-Being

Well-being was measured across 11 domains, based on the OECD Better Life Index. The domains were as follows: housing, income, jobs, community, education, environment, governance, health, life satisfaction, safety, and work–life balance. Each item was scored on an 11-point scale (0 = “not at all satisfied” to 10 = “very satisfied”).

#### 2.2.3. Covariates

We included demographic and socioeconomic covariates as follows: age, sex, urbanicity, household size, work status, housing type, total floor area, health status, education, annual household income, financial assets, and debt.

### 2.3. Statistical Analysis

Descriptive statistics were calculated for all variables. Chi-square tests were used to compare demographic characteristics by pet ownership status. Linear regression analyses were performed to examine associations between pet ownership and well-being outcomes. Model 1 included only the pet ownership, while Model 2 adjusted for all covariates. For each model, we report coefficients, 95% confidence intervals, and *p*-values. All analyses were conducted using R version 4.4.2. A *p*-value of <0.05 was considered statistically significant.

## 3. Results

### 3.1. Descriptive Statistics

Of the total 10,293 participants, 13.3% reported owning a co-walkable pet such as a dog, 13.0% owned an indoor-only pet such as a cat, and 6.8% owned an ornamental pet such as a tropical fish. Approximately 70.0% (*n* = 7210) reported not owning these pets (‘no’ to all questions for co-walkable pet, indoor-only pet, and ornamental pet). Participants had multiple pets of different types were: 1.7% (*n* = 177) for co-walkable type and indoor type, 1.0% (*n* = 100) for co-walkable type and ornamental type, 0.8% (*n* = 83) for indoor pet and ornamental type, and 0.3% (*n* = 36) for all the three types. As shown in Table 1, chi-square tests revealed significant differences between owners and non-owners for each pet type across multiple sociodemographic variables:

- For co-walkable pets, significant differences were observed in age, urbanicity, household size, housing type, total floor area, education, annual household income, financial assets, and debt.
- For indoor-only pets, significant differences were found in age, urbanicity, household size, work status, housing type, total floor area, health status, education, annual household income, and debt.
- For ornamental pets, significant differences were detected in age, sex, household size, work status, housing type, total floor area, annual household income, financial assets, and debt.

**Table 1:** Demographic characteristics according to three types of pet ownership.

		Total		Co-Walkable Pet (e.g., Dog)								Indoor Pet (e.g., Cat)								Ornamental Pet (e.g., Fish)							
				with		Without						with		Without						with		Without					
		*N* = 10,293		*N* = 1369		*N* = 8924						*N* = 1339		*N* = 8954						*N* = 699		*N* = 9594					
		(100%)		(13.3%)		(86.7%)						(13.0%)		(87.0%)						(6.8%)		(93.2%)					
		*n*	%	*n*	%	*n*	%	χ^2^	df	*p*		*n*	%	*n*	%	χ^2^	df	*p*		*n*	%	*n*	%	χ^2^	df	*p*	
Age								6.68	2	0.041	*					11.67	2	0.001	**					9.39	2	0.011	*
	<40	4565	44.4	568	41.5	3997	44.8					572	42.7	3993	44.6					318	45.5	4247	44.3				
	<60	3151	30.6	456	33.3	2695	30.2					462	34.5	2689	30.0					238	34.0	2913	30.4				
	≥60	2577	25.0	345	25.2	2232	25.0					305	22.8	2272	25.4					143	20.5	2434	25.4				
Sex								2.95	1	0.091	†					3.24	1	0.071	†					11.1	1	<0.001	***
	Male	5102	49.6	649	47.4	4453	49.9					633	47.3	4469	49.9					389	55.7	4713	49.1				
	Female	5191	50.4	720	52.6	4471	50.1					706	52.7	4485	50.1					310	44.3	4881	50.9				
Urbanicity								20.68	2	<0.001	***					7.0	2	0.031	*					3.96	2	0.141	
	≥1 million people	1571	15.3	164	12.0	1407	15.8					190	14.2	1381	15.4					96	13.7	1475	15.4				
	≥0.1 million people	5586	54.3	730	53.3	4856	54.4					700	52.3	4886	54.6					368	52.6	5218	54.4				
	<0.1 million people	3136	30.5	475	34.7	2661	29.8					449	33.5	2687	30.0					235	33.6	2901	30.2				
Household size								94.29	1	<0.001	***					31.12	1	<0.001	***					41.99	1	<0.001	***
	Living alone	1643	16.0	96	7.0	1547	17.3					144	10.8	1499	16.7					51	7.3	1592	16.6				
	Two or more	8650	84.0	1273	93.0	7377	82.7					1195	89.2	7455	83.3					648	92.7	8002	83.4				
Work status								2.00	1	0.161						6.5	1	0.011	*					4.4	1	0.041	*
	Not working (including students)	3192	31.0	402	29.4	2790	31.3					375	28.0	2817	31.5					192	27.5	3000	31.3				
	Working	7101	69.0	967	70.6	6134	68.7					964	72.0	6137	68.5					507	72.5	6594	68.7				
Housing type								194.85	1	<0.001	***					42.61	1	<0.001	***					34.01	1	<0.001	***
	Not owned	3322	32.3	217	15.9	3105	34.8					328	24.5	2994	33.4					156	22.3	3166	33.0				
	Owned	6971	67.7	1152	84.1	5819	65.2					1011	75.5	5960	66.6					543	77.7	6428	67.0				
Total floor area								108.54	4	<0.001	***					23.34	4	<0.001	***					16.38	4	0.001	**
	<25 m^2^	931	9.0	58	4.2	873	9.8					79	5.9	852	9.5					38	5.4	893	9.3				
	<50 m^2^	2405	23.4	243	17.8	2162	24.2					307	22.9	2098	23.4					149	21.3	2256	23.5				
	<100 m^2^	3341	32.5	451	32.9	2890	32.4					443	33.1	2898	32.4					238	34.0	3103	32.3				
	<150 m^2^	2066	20.1	337	24.6	1729	19.4					276	20.6	1790	20.0					154	22.0	1912	19.9				
	≥150 m^2^	1550	15.1	280	20.5	1270	14.2					234	17.5	1316	14.7					120	17.2	1430	14.9				
Health status								0.11	1	0.741						5.48	1	0.021	*					1.33	1	0.251	
	Not good	2193	21.3	287	21.0	1906	21.4					318	23.7	1875	20.9					161	23.0	2032	21.2				
	Good	8100	78.7	1082	79.0	7018	78.6					1021	76.3	7079	79.1					538	77.0	7562	78.8				
Education								18.32	2	<0.001	***					26.99	2	<0.001	***					1.56	2	00.461	
	Middle or high school	3388	32.9	507	37.0	2881	32.3					520	38.8	2868	32.0					245	35.1	3143	32.8				
	Vocational school, technical college, junior college	2392	23.2	332	24.3	2060	23.1					305	22.8	2087	23.3					156	22.3	2236	23.3				
	University, graduate school	4513	43.8	530	38.7	3983	44.6					514	38.4	3999	44.7					298	42.6	4215	43.9				
Annual household income								85.86	4	<0.001	***					13.45	4	0.011	*					16.32	4	00.001	**
	JPY < 3 million	2538	24.7	267	19.5	2271	25.4					297	22.2	2241	25.0					133	19.0	2405	25.1				
	JPY < 5 million	2773	26.9	335	24.5	2438	27.3					336	25.1	2437	27.2					183	26.2	2590	27.0				
	JPY < 7 million	2196	21.3	267	19.5	1929	21.6					316	23.6	1880	21.0					171	24.5	2025	21.1				
	JPY < 10 million	1713	16.6	279	20.4	1434	16.1					229	17.1	1484	16.6					128	18.3	1585	16.5				
	JPY ≥ 10 million	1073	10.4	221	16.1	852	9.5					161	12.0	912	10.2					84	12.0	989	10.3				
Financial assets								18.51	3	<0.001	***					7.48	3	0.061	†					19.83	3	<0.001	***
	JPY < 1 million	2488	24.2	281	20.5		2207					335	25.0		2153					121	17.3		2367				
	JPY < 5 million	3237	31.4	416	30.4		2821					378	28.2		2859					246	35.2		2991				
	JPY < 20 million	3070	29.8	439	32.1		2631					420	31.4		2650					227	32.5		2843				
	JPY ≥ 20 million	1498	14.6	233	17.0		1265					206	15.4		1292					105	15.0		1393				
Debt								32.26	1	<0.001	***					6.35	1	0.011	*					28.88	1	<0.001	***
	JPY < 1 million	6816	66.2	814	59.5	6002	67.3					846	63.2	5970	66.7					398	56.9	6418	66.9				
	JPY ≥ 1 million	3477	33.8	555	40.5	2922	32.7					493	36.8	2984	33.3					301	43.1	3176	33.1				

*** *p* < 0.001; ** *p* < 0.01; * *p* < 0.05; † *p* < 0.10.

### 3.2. Association Between Pet Ownership and Well-Being (Unadjusted Models)

In the unadjusted linear regression models (Table 2), the ownership of co-walkable pets was positively associated with well-being in the domains of housing (*β* = 0.16, 95%CI = 0.03–0.29, *p* = 0.03) and community (*β* = 0.15, 95%CI = 0.03–0.27, *p* = 0.016). The ownership of indoor-only or ornamental pets was not significantly associated with any well-being outcomes in the unadjusted models.

### 3.3. Association Between Pet Ownership and Well-Being (Adjusted Models)

After adjusting for demographic and socioeconomic covariates (Table 2), dog ownership was negatively associated with well-being in the domains of income (*β* = −0.12, 95%CI = −0.24–0.00, *p* = 0.044), jobs (*β* = −0.12, 95%CI = −0.24–0.00, *p* = 0.043), and environment (*β* = −0.15, 95%CI = −0.26–−0.04, *p* = 0.007). A marginal association was also observed for safety (*β* = −0.10, 95%CI = −0.21–0.01, *p* = 0.067). Cat and fish ownership was not significantly associated with any well-being domains in the adjusted models.

## 4. Discussion

This study examined the association between pet ownership and subjective well-being in Japan, a non-Western cultural context, using a large, nationally representative dataset. Pet ownership was categorized into three types: co-walkable pets (e.g., dogs), indoor-only pets (e.g., cats), and ornamental pets (e.g., tropical fish). Well-being was assessed across eleven domains based on the OECD Better Life Index. Contrary to the generally positive expectations, our findings presented a more nuanced picture. The ownership of indoor-only and ornamental pets showed no significant associations with any well-being domains after adjusting for covariates. Surprisingly, the ownership of co-walkable pets was associated with lower well-being in specific domains, such as income, jobs, and environmental satisfaction. Given the different types and levels of interaction with humans, it is meaningful to categorize pets into co-walkable, indoor-only, and ornamental types. Furthermore, well-being itself is not a monolithic concept. Although life satisfaction is a frequently used global measure, well-being consists of multiple domains such as income, job satisfaction, environmental satisfaction, health perception, and more, each of which may be differentially associated with pet ownership.

Pet ownership encompasses a wide variety of animals with different levels of physical and emotional interactions with humans. Although many prior studies have reported positive associations between pet ownership and well-being, the so-called “pet effect” remains inconclusive. As identified in a recent systematic review, five studies reported negative associations, including increased depression or loneliness [6]. For instance, a large-scale nationally representative study from Sweden (*n*= 39,995) found that pet owners reported more mental health problems and physical pain than non-owners [14]. Another study conducted among older adults in Australia (*n*= 2551) unexpectedly found higher levels of psychoticism among pet owners [15]. In a survey of Australian pet owners (*n*= 150), psychological distress was positively associated with stronger emotional attachment to pets [16]. Similarly, research among Canadian individuals living alone (*n*= 132) found that perceived social support from humans, rather than pets, had a greater impact on loneliness [17]. Taken together, these findings suggest that reverse causality may partially explain our results: individuals with lower well-being or mental health challenges may be more likely to adopt pets, particularly in the absence of human social support.

Dogs are one of the most popular pets and have been widely studied in relation to both their physical and mental health benefits. Several studies have suggested that dog walking contributes to reduced sedentary time, increased physical activity, and enhanced community connectedness [1,18,19]. These behavioral aspects may, in turn, contribute to improved well-being. In our cross-sectional study, the results show a correlation, not causation, and thus cannot be used to determine whether the owners of co-walkable pets were more likely to report low well-being or people with low well-being were more likely to own a co-walkable pet. Additionally, the negative associations observed for co-walkable pet ownership in the adjusted models may indicate that pets are not sufficient to mitigate dissatisfaction rooted in socioeconomic factors. Another possible explanation is that walking a pet increases one’s exposure to neighborhood conditions, which may heighten awareness of environmental or safety concerns. Also, there might be a connection with pet-friendly buildings and public spaces. Previous research revealed that dog ownership in Japan was negatively associated with walkability in neighborhoods [20]. Dog owners may live in pet-friendly houses or apartments, but be surrounded by a non-walkable environment, and end up with dissatisfaction in this regard. In terms of income, the pet-related expenditure was considerable, showing an increase recently [21,22]. The financial burden caused by pet ownership may interfere with the owner’s economic satisfaction. In terms of jobs, a scoping review reported that workplaces with pets have the ability to reduce stress but also increase distraction and interruptions [23]. The results of pets’ effects on work seem to be also mixed. Regarding different types of pets, previous research on the ownership of pets including dogs, cats, fishes, and other animals revealed that owning pets other than dogs was not associated with mortality [24]. The association of dog ownership and mortality was shown to be mediated by physical activity. The association between pet ownership and well-being in this study might also be determined by the owner’s activity level.

Indoor-only pets, such as cats, do not require co-walking, but may still provide psychological comfort through tactile interaction (e.g., petting) and companionship, as well as a sense of responsibility and affection often attributed to family members. In our study, the disappearance of marginal negative associations between indoor-only pet ownership and well-being after covariate adjustment suggests that demographic factors—rather than pet ownership itself—may account for the observed differences. In limited studies, as reported by a systematic review, ornamental pets such as tropical fish, though lacking direct physical contact, have been shown to reduce stress through visual engagement, potentially supporting mental relaxation [9]. However, no significant association has been confirmed in our study.

This study has several notable strengths. It utilized a large, population-based sample with national representativeness, and employed well-established, internationally comparable indicators of well-being based on the OECD Better Life Index (https://doi.org/10.34500/SSJDA.1423, accessed on 19 May 2025). It also distinguished between pet types, including ornamental pets, which have often been overlooked in previous research. Furthermore, commonly known covariates of well-being were thoroughly controlled. However, this study also has limitations. The dataset did not distinguish pet ownership from caregiving responsibilities or emotional attachment to animals. The pet categories used may not perfectly correspond to specific species, and information on the participants’ ethnicity was unavailable. Most importantly, the cross-sectional design precludes any causal inference. Future research using longitudinal or mixed-method designs, including additional psychological and behavioral variables, may offer a more comprehensive understanding of the complex relationship between humans and companion animals.

## 5. Conclusions

This study provides one of the first nationally representative analyses of the relationship between pet ownership and subjective well-being in Japan, distinguishing between co-walkable, indoor-only, and ornamental pets. The results challenge the conventional notion of a universally positive “pet effect” by demonstrating that, after adjusting for demographic and socioeconomic factors, owners of co-walkable pets such as dogs reported lower satisfaction in economic and environmental domains. These findings suggest that pet ownership does not necessarily enhance well-being and may, in some cases, reflect underlying socioeconomic or environmental challenges. Nevertheless, this study’s cross-sectional design limits causal interpretation, and information regarding emotional attachment or specific caregiving involvement was unavailable. Future longitudinal or qualitative studies should examine the mechanisms underlying pet ownership, socioeconomic factors, and well-being outcomes more closely. Further research integrating psychological, behavioral, and environmental variables could deepen our understanding of the diverse roles of companion animals in human lives. Such insights may inform more balanced, context-sensitive policies and interventions related to animal companionship and human well-being.

## Figures and Tables

**Table 2:** Results of linear regression analysis of well-being score from pet ownership.

		Co-Walkable Pet (e.g., Dog)						Indoor Pet (e.g., Cat)						Ornamental Pet (e.g., Fish)				
		*β*	95%CI			*p*		*β*	95%CI			*p*		*β*	95%CI			*p*
Housing																		
	Unadjusted	0.16	0.03–0.29			0.014	*	−0.02	−0.15–0.11			0.791		0.11	−0.07–0.28			0.222
	Adjusted	−0.07	−0.19–0.05			0.266		−0.05	−0.17–0.07			0.407		0.03	−0.13–0.19			0.738
Income																		
	Unadjusted	0.02	−0.12–0.15			0.793		−0.13	−0.27–0.00			0.058	†	−0.01	−0.19–0.17			0.925
	Adjusted	−0.12	−0.24–0.00			0.044	*	−0.10	−0.22–0.02			0.102		−0.04	−0.20–0.12			0.651
Jobs																		
	Unadjusted	−0.02	−0.15–0.11			0.789		−0.08	−0.21–0.05			0.212		0.05	−0.12–0.22			0.554
	Adjusted	−0.12	−0.24–0.00			0.043	*	−0.05	−0.17–0.07			0.388		0.04	−0.12–0.20			0.638
Community																		
	Unadjusted	0.15	0.03–0.27			0.016	*	−0.07	−0.19–0.05			0.238		0.06	−0.10–0.22			0.456
	Adjusted	0.06	−0.05–0.17			0.305		−0.05	−0.16–0.07			0.403		0.08	−0.07–0.23			0.309
Education																		
	Unadjusted	0.02	−0.10–0.14			0.718		−0.11	−0.23–0.01			0.083	†	−0.01	−0.17–0.15			0.891
	Adjusted	−0.02	−0.13–0.09			0.732		−0.02	−0.13–0.09			0.693		0.03	−0.12–0.17			0.713
Environment																		
	Unadjusted	−0.07	−0.19–0.04			0.207		0.03	−0.09–0.14			0.645		−0.03	−0.19–0.12			0.692
	Adjusted	−0.15	−0.26–−0.04			0.007	**	0.05	−0.06–0.16			0.360		−0.05	−0.20–0.10			0.534
Governance																		
	Unadjusted	−0.04	−0.16–0.08			0.490		−0.06	−0.18–0.06			0.316		0.12	−0.04–0.28			0.134
	Adjusted	−0.10	−0.21–0.02			0.101		−0.03	−0.15–0.08			0.581		0.12	−0.04–0.27			0.132
Health																		
	Unadjusted	0.04	−0.09–0.16			0.568		−0.05	−0.17–0.08			0.472		−0.07	−0.23–0.11			0.455
	Adjusted	−0.03	−0.14–0.07			0.525		0.03	−0.08–0.14			0.583		−0.03	−0.17–0.11			0.662
Life satisfaction																		
	Unadjusted	0.09	−0.05–0.22			0.192		−0.12	−0.25–0.02			0.087	†	0.00	−0.18–0.18			0.964
	Adjusted	−0.04	−0.16–0.09			0.542		−0.09	−0.22–0.03			0.147		0.00	−0.16–0.17			0.972
Safety																		
	Unadjusted	−0.04	−0.16–0.07			0.466		−0.02	−0.13–0.10			0.774		0.01	−0.14–0.16			0.886
	Adjusted	−0.10	−0.21–0.01			0.067	†	0.02	−0.09–0.13			0.676		0.02	−0.13–0.16			0.832
Work–life balance																		
	Unadjusted	0.08	−0.04–0.20			0.208		−0.04	−0.16–0.09			0.587		0.01	−0.16–0.18			0.920
	Adjusted	−0.03	−0.15–0.09			0.618		−0.01	−0.13–0.10			0.831		0.01	−0.15–0.16			0.941

** *p* < 0.01; * *p* < 0.05; † *p* < 0.10.

## Data Availability

The data for this secondary analysis were provided by the Social Science Japan Data Archive, Center for Social Research and Data Archives, Institute of Social Science, The University of Tokyo.

## Abbreviations

The following abbreviations are used in this manuscript:

OECD	Organization for Economic Co-operation and Development
CI	Confidence interval
